# Electroencephalography in emerging viral infections: Lessons learned from implementing an EEG unit in a Lassa fever isolation ward in Nigeria

**DOI:** 10.1371/journal.pntd.0012522

**Published:** 2024-10-02

**Authors:** Hannah Caroline Sophie Mueller, Cyril Oshomah Erameh, Mathias Gelderblom, Osahogie Isaac Edeawe, Osetohamen Grace Akpasubi, Ekpen Uwayeme Ekoyata, Ujiagbe Moses Aiterebhe, Joseph Okoeguale, Stephan Guenther, Lisa Oestereich, Michael Ramharter, Sylvanus Okogbenin, Till Omansen

**Affiliations:** 1 Department of Tropical Medicine, Bernhard Nocht Institute for Tropical Medicine & I. Department of Medicine, University Medical Center Hamburg-Eppendorf, Hamburg, Germany; 2 Department of Virology, Bernhard Nocht Institute for Tropical Medicine, Hamburg, Germany; 3 Institute of Viral and Emergent Pathogens Control and Research, Irrua Specialist Teaching Hospital, Irrua, Nigeria; 4 Department of Medicine, Irrua Specialist Teaching Hospital, Irrua, Nigeria; 5 Department of Neurology, University Medical Center Hamburg-Eppendorf, Hamburg, Germany; 6 Department of Pharmacology and Therapeutics, Ambrose Alli University, Ekpoma, Nigeria; 7 Department of Obstetrics and Gynecology, Irrua Specialist Teaching Hospital, Irrua, Nigeria; 8 German Center for Infection Research, Partner Sites Hamburg-Lübeck-Borstel-Riems, Germany; Public Health Agency of Canada, CANADA

## Abstract

Electroencephalography (EEG) has been used for almost a century in well-equipped medical centers to facilitate the diagnosis of epilepsy and other brain disorders. Lassa fever (LF) and other emerging viral infections (EVI) are known to cause neurological complications, including meningitis, seizures, and encephalopathy, though to date it remains unclear whether these are secondary to metabolic disturbances caused by the disease or by direct involvement of the central nervous system (CNS). To better characterize how Lassa virus (LASV) affects the CNS, we established an EEG diagnostic unit in the LF isolation ward at Irrua Specialist Teaching Hospital in Edo State, Nigeria. Here, we report on the specific difficulties to successful implementation of EEG in this highly challenging setting. Technical artefacts due to electrical interferences and interrupted power supply, artefacts deriving from a partly improvised EEG setup within a high consequence pathogen isolation ward, and environmental factors, such as heat in the endemic West African setting are among the main difficulties encountered when setting up this diagnostic facility. It takes experienced neurophysiologists to distinguish such artefacts from actual EEG abnormalities as many of them are not commonly encountered to this extent in well-equipped EEG laboratories and can easily be confused with pathologies. The EEG recording process is further complicated by biosafety considerations and the necessity of wearing extensive personal protective equipment. Nevertheless, with the help of experienced neurophysiologists, it is possible to correctly set up the facility and interpret recordings. Taking the above into consideration, EEG is valuable in identifying CNS involvement in emerging infections, particularly regarding assessment of encephalitis, differential diagnosis of impaired consciousness and treatment adjustment in patients with symptomatic seizures. Although highly challenging under these circumstances, EEG can be an important, noninvasive diagnostic tool for neurological complications in EVI where other more advanced imaging modalities are not available.

## Author summary

Lassa fever (LF) is an emerging viral infection which, in severe cases, may result in viral hemorrhagic fever with neurological complications like meningitis, confusion, and seizures. Electroencephalography (EEG), a well-established diagnostic tool for neurological diseases, measures the electric activity of the brain. It helps to differentiate between various causes of an altered level of consciousness and seizures and is essential to guide anti-convulsive therapy. We established an EEG unit at the LF isolation ward of Irrua Specialist Teaching Hospital in Nigeria, which is the worldwide largest LF treatment center. In emerging viral infections like LF, EEG can be a valuable diagnostic tool in the absence of neuroimaging to detect cerebral involvement. In viral encephalitis, EEGs typically show general slowing of the background activity, encephalopathic patterns and epileptic seizures. Establishing EEG in a resource-limited and high consequence pathogen isolation ward setting requires attention to unique technical pitfalls not commonly observed in well-equipped medical centers. To prevent misinterpretation due to technical artefacts, EEG setup and interpretation should be overseen by experienced neurophysiologists. With these challenges in mind, EEG presents a vital diagnostic tool for neurological complications in emerging viral infections.

## Viewpoint

Electroencephalography (EEG) is a noninvasive diagnostic tool which measures the electrical activity of the cerebral cortex. It has been used for almost a century [1] in well-equipped medical centers to diagnose neurological disorders such as unexplained altered level of consciousness, seizures, and focal deficits.

Numerous viral agents are known to affect the central nervous system (CNS), such as herpes simplex virus [2,3], varicella zoster virus [3], human immunodeficiency virus [3], tick-borne encephalitis virus [4], Japanese encephalitis virus [5], and Ebola virus [6]. The same is suspected of Lassa virus (LASV), which has been isolated from cerebrospinal fluid (CSF) of affected patients [7,8] and causes meningitis, seizures, and encephalopathy [9]. Neurological complications in Lassa fever (LF) are strongly associated with fatal disease outcome [10,11]. Viruses may affect the CNS by various mechanisms, such as inflammation, bleeding, or direct neurotropic effects [12,13], though it remains unclear which pathomechanism LASV exerts.

We established a dedicated EEG diagnostic unit in the viral hemorrhagic fever (VHF) isolation ward at Irrua Specialist Teaching Hospital, the largest LF treatment center globally, located in a high transmission region of Nigeria. We argue that in resource-limited and isolation ward settings, EEG, if carefully set up and applied, is a clinically useful diagnostic modality to identify neurological complications in emerging viral infections (EVI), including VHF. However, there are significant challenges and pitfalls in both EEG recording and interpretation in such settings that need to be considered.

### Useful applications of electroencephalography (EEG)

EEG is an inexpensive, fast, and noninvasive diagnostic tool that depicts the electrical activity of the brain with high-temporal resolution. Although its spatial resolution is poor, EEG is valuable in contexts where neuroimaging is unavailable as it may generate meaningful findings in both epilepsy and encephalitis and is therefore ideal for use in highly infectious patients and in resource-limited settings, as in VHF. It takes approximately 20 min preparation and recording time, respectively and a trained neurophysiologist can analyze an EEG in 5 min.

Beyond its essential role in diagnosing seizures and (nonconvulsive) status epilepticus, EEG is used to differentiate between various causes of impaired consciousness, such as meningoencephalitis, stroke, metabolic encephalopathies, cerebral edema, or drug and medication intake. In case of emerging viral and other infections, EEG can identify CNS involvement. EEG abnormalities found in viral encephalitis include generalized slowing, as in West Nile virus neuroinvasive disease [14] and tick-borne encephalitis [4]. Diffuse delta activity occurs in Japanese encephalitis, at times associated with spike-and-wave discharges and apparent side asymmetries [5], and in herpes encephalitis [2]. The latter also causes focal slowing in the temporal area [3], often accompanied by lateralized periodic discharges, intermittent generalized rhythmic delta activity, and both ictal and interictal EEG patterns [2]. CNS involvement of Puumala Hantavirus infection, which like LF is a Bunyavirus infection and may manifest as VHF, leads to mild generalized slowing and intermittent rhythmic delta activity in the EEG [15].

**Fig 1:**
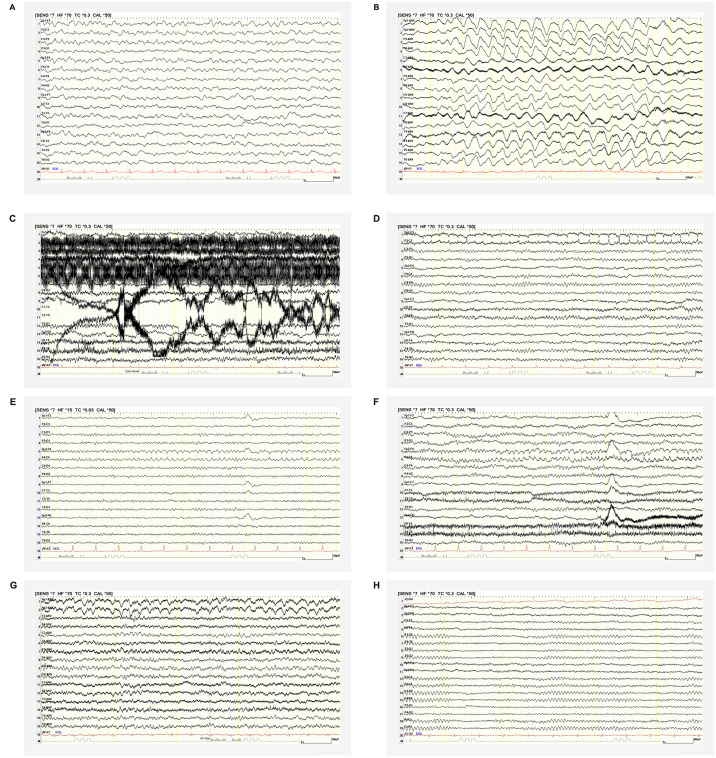
Typical EEG findings in Lassa fever patients and misinterpretation pitfalls in the resource-limited LF isolation ward setting. (A) EEG of a 32-year-old male LF patient showing theta frequency background slowing (6 Hz) with reversed anterior-posterior gradient. (B) Typical intermittent, high-amplitude, frontally predominant, 2.5 Hz generalized rhythmic delta activity in the EEG of a 31-year-old male patient. (C) Severe artefact overlay (muscle artefacts in T4 and T6, T3 electrode artefacts and electrical disturbances in the frontal electrodes) make this otherwise normal EEG largely unreadable. (D) F3 electrode artefact which can be confused with focal epileptiform discharges as seen in patients with focally triggered seizures as a sign of increased seizure susceptibility. (E, F) Same EEG section with incorrect (E) and correct (F) time constant and high-frequency filter settings. Incorrect filter settings simulate better recording quality by masking certain artefacts: A falsely reduced time constant of 0.03 seconds masks sub-delta activity, like sweat artefacts (C3, P3, and F4), by smoothing the EEG baseline. A high-frequency filter incorrectly lowered to 15 Hz reduces high-amplitude electrode artefacts, promoting a potential misinterpretation of epileptiform potentials as demonstrated by the F4 electrode artefact. (G) Eyelid-related eye artefacts can be mistaken for bifrontal delta activity in the absence of EOG. (H) EEG without abnormalities in appropriate recording quality after numerous adjustments of our EEG setup, correct filter setting application (time constant 0.3 s; high-frequency filter 70 Hz), and EOG addition.

Common EEG pathologies observed in our LF patients were EEG background slowing (Fig 1A) and generalized rhythmic delta activity (Fig 1B), which may occur in both viral encephalitis and metabolic encephalopathy and therefore need to be interpreted in the overall context of clinical and laboratory parameters. Focal slowing of the EEG activity seen in our LF patients points towards a localized cerebral pathology, such as cerebral hemorrhage or encephalitis, and requires further examination. Increased seizure susceptibility after an observed seizure warrants either the start of anticonvulsive therapy or adjustment of an existing treatment to prevent further convulsions.

### Challenges of EEG implementation in a resource-limited high consequence pathogen isolation ward

The EEG implementation in an isolation ward of a LASV-endemic setting poses unique challenges which result from artefacts not commonly encountered in well-equipped medical centers due to a partly improvised EEG setup and environmental factors. Electric artefacts resulting from a lack of electric shielding of the EEG room and interrupted power supply compromise the recording quality (Fig 1C). Muscle artefacts may occur when patients are positioned on upright chairs, resulting in poorer muscle relaxation (Fig 1C). Electrode artefacts may arise if unsuitable consumable materials such as skin scrub and electrode pastes are used and when electrodes and cables wear out faster under difficult storage conditions of high air temperature and humidity (Fig 1D). The latter also cause significant sweat artefacts in recordings without adequate temperature control of the EEG chamber (Fig 1F). Without electrooculography (EOC) installed to record eye activity, artefacts caused by eye movement or eyelid fluttering may resemble brain inherent activity (Fig 1G).

These limitations in recording quality create interpretation pitfalls, particularly when incorrect filter settings are applied, such as a falsely reduced high-frequency filter to compensate for muscle artefacts (see Fig 1E and 1F).

Fig 1D demonstrates an F3 electrode artefact which can be confused with focal epileptiform discharges (spikes) as seen in patients with focally triggered seizures as sign of increased seizure susceptibility. However, the negative phase reversal and the fact that the potential was only detectable under F3 expose this as an artefact. Fig 1E and 1F illustrate how an F4 electrode artefact may be mistaken for a focal seizure correlate when incorrect EEG filter settings are applied, as it shows a rhythmic fire pattern with typical characteristics of ictal activity in the EEG: a slow increase in amplitude and decrease in frequency over time. A lowered high-frequency filter cuts of the EEG potentials’ amplitude peaks, thereby reducing high-amplitude electrode or motion artefacts. This results in an overall flattening of EEG signals, however, which may cause epileptiform potentials, defined as spiky or sharp, to be overlooked. Additionally, incorrect filter settings influence the morphology of potentials, so that artefacts may be misinterpreted as epileptiform potentials. Only correct filter settings allow to clearly identify artefacts, such as the F4 artefact shown here, even if the overall EEG quality is limited.

Due to the abovementioned pitfalls, involving experienced neurophysiologists in EEG setup, staff training and interpretation is important to ensure accurate results.

Fig 1H shows an EEG without abnormalities in appropriate recording quality after substantial adjustments of our EEG setup, application of correct filter settings of time constant (0.3 s) and high-frequency filter (70 Hz) as per internationally standardized EEG recording criteria and EOG addition.

Apart from these difficulties, biosecurity considerations present another important challenge when implementing EEG for EVI patients. The mounting of the EEG cap and placement of electrodes, which involve close contact with the patient’s head, a potential risk for virus transmission, require considerable tactile sensitivity and are complicated by the extensive personal protective equipment necessary when working with VHF pathogens.

## Conclusions

EEG is a useful diagnostic tool to assess cerebral involvement in EVI patients presenting with neurological symptoms when results are placed in the context of clinical and laboratory parameters, particularly where neuroimaging is unavailable. Specifically, EEG should be applied in case of seizures to estimate their recurrence risk, guide antiepileptic treatment, and for the differential diagnosis of encephalopathy and impaired consciousness.

The EEG setup needs to be controlled thoroughly to ensure adequate recording quality and prevent artefacts (Fig 1F), when placed in a resource-limited isolation ward setting. In the establishment process, EEGs should be interpreted with the help of experienced neurophysiologists for quality assurance. Due to biosafety considerations, particular caution is required when mounting the EEG cap in extensive personal protective equipment as necessary in the care of VHF patients.

In summary, more research is necessary to advance the understanding of neurological complications of LF. Besides application in humans, where EEG contributes to epidemic preparedness by adding a fast diagnostic tool, it might also be useful in VHF studies with nonhuman primates.
